# Virus expression detection reveals RNA-sequencing contamination in TCGA

**DOI:** 10.1186/s12864-020-6483-6

**Published:** 2020-01-28

**Authors:** Sara R. Selitsky, David Marron, Daniel Hollern, Lisle E. Mose, Katherine A. Hoadley, Corbin Jones, Joel S. Parker, Dirk P. Dittmer, Charles M. Perou

**Affiliations:** 1 Lineberger Comprehensive Cancer Center, University of North Carolina at Chapel Hill, Chapel Hill, North Carolina 27599 USA; 2 Department of Genetics, University of North Carolina at Chapel Hill, Chapel Hill, North Carolina 27599 USA; 3 Department of Biology, University of North Carolina at Chapel Hill, Chapel Hill, North Carolina USA; 4 Department of Microbiology and Immunology, University of North Carolina at Chapel Hill, Chapel Hill, North Carolina USA

**Keywords:** Virus detection, Bioinformatics, Contamination, Human papilloma virus, Xenotropic murine leukemia virus-related

## Abstract

**Background:**

Contamination of reagents and cross contamination across samples is a long-recognized issue in molecular biology laboratories. While often innocuous, contamination can lead to inaccurate results. Cantalupo et al.*,* for example, found HeLa-derived human papillomavirus 18 (H-HPV18) in several of The Cancer Genome Atlas (TCGA) RNA-sequencing samples. This work motivated us to assess a greater number of samples and determine the origin of possible contaminations using viral sequences. To detect viruses with high specificity, we developed the publicly available workflow, VirDetect, that detects virus and laboratory vector sequences in RNA-seq samples. We applied VirDetect to 9143 RNA-seq samples sequenced at one TCGA sequencing center (28/33 cancer types) over 5 years.

**Results:**

We confirmed that H-HPV18 was present in many samples and determined that viral transcripts from H-HPV18 significantly co-occurred with those from xenotropic mouse leukemia virus-related virus (XMRV). Using laboratory metadata and viral transcription, we determined that the likely contaminant was a pool of cell lines known as the “common reference”, which was sequenced alongside TCGA RNA-seq samples as a control to monitor quality across technology transitions (i.e. microarray to GAII to HiSeq), and to link RNA-seq to previous generation microarrays that standardly used the “common reference”. One of the cell lines in the pool was a laboratory isolate of MCF-7, which we discovered was infected with XMRV; another constituent of the pool was likely HeLa cells.

**Conclusions:**

Altogether, this indicates a multi-step contamination process. First, MCF-7 was infected with an XMRV. Second, this infected cell line was added to a pool of cell lines, which contained HeLa. Finally, RNA from this pool of cell lines contaminated several TCGA tumor samples most-likely during library construction. Thus, these human tumors with H-HPV or XMRV reads were likely not infected with H-HPV 18 or XMRV.

## Background

Rigorous and reproducible experiments should minimize extrinsic factors that could bias the results. Nevertheless, contamination in molecular biology is a well-described problem [1]. Here we investigated the source(s) of viral contamination in The Cancer Genome Atlas (TCGA) pan-cancer RNA-seq dataset. The two types of contamination that were uncovered in this study were (a) unexpected viral infection of a cell line and (b) unexpected contamination of massively parallel sequencing experiments. A previous example of an unexpected viral contamination was the discovery of a xenotropic murine leukemia virus-related virus (XMRV) in the human prostate cancer cell line, 22Rv1 [2–4]. After this initial discovery, other strains of XMRVs have been found in additional cell lines [5–7]. These include both complete and defective proviral genomes. Some XMRVs make infectious particles and thus have the ability to infect other cell lines in culture. Yet, infection does not cause overt phenotypes. This can lead to an unnoticeable contamination of cell lines in culture.

The other type of contamination uncovered in this study was contamination during the sequencing process [1, 8–11]. The sensitivity of sequencing technology allows for minimal amounts of contaminating nucleic acids to manifest in the data. Ballenghien et al. found 80% of samples from a large-scale sequencing experiment had evidence of cross-contamination, which they demonstrated likely occurred in the sequencing center [1]. Robinson et al. demonstrated that bacterial species detected from RNA and DNA sequencing were associated with specific sequencing centers in TCGA, indicating possible contamination [10]. Finally, HeLa-derived human papillomavirus 18 (H-HPV18) was discovered in non-cervical cancer samples in TCGA RNA-seq [11]. This motivated us to test the extent and origin of H-HPV18 contamination, as well as other possible viral sequences in the RNA-seq from TCGA. We investigate contamination through association with laboratory processing variables including time of sequence generation and laboratory controls. To assess the contamination, we created the virus detection software, VirDetect.

## Results

### A highly specific virus detection software: VirDetect

**Fig. 1:**
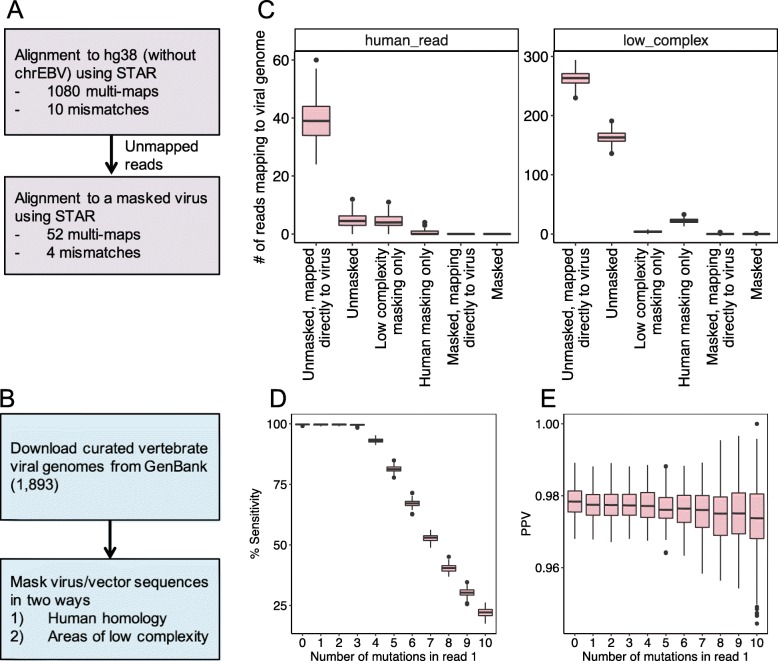
VirDetect workflow and performance. **a** & **b** VirDetect workflow diagram **a** VirDetect alignment steps, **b** virus genome preparation steps. **c** Number of reads mapping to the viral genome for both human (left) and low complexity (right) simulated reads (100 simulated samples, with 1000,000 human reads and 1000 low complexity reads each). From left to right on x-axis: (1) Unmasked, directly to the virus: all reads directly mapped to the unmodified viral genomes, without filtering human reads. (2) Unmasked: reads unaligned to the human genome were aligned to the unmodified viral genomes. (3) Low complexity masking only: reads unaligned to the human genome were aligned to the viral genomes masked for areas of low complexity. (4) Human masking only: reads unaligned to the human genome were aligned to viral genomes that were masked in areas of human homology. (5) Masked, mapping directly to the virus: all reads were mapped directly to the masked viral genomes, without filtering reads out that map the human genome. (6) Masked: reads unaligned to the human genome were aligned to masked viral genomes. **d** & **e** Viral simulated reads (100 simulated samples with 1000 reads each) with 0–10 mutations in the first read pair (**d**) Sensitivity, measured by the percent of reads that mapped to the viral genomes. **e** Positive predictive value (PPV) measured by number of true positives (simulated viral reads that mapped to the correct viral genomes) divided by the number of true positives and false positives

To detect viruses from RNA-seq data, we developed VirDetect, an open source software based on the principles of digital subtraction [12–16]. VirDetect begins by aligning RNA-seq reads to the human genome using the STARv2.4 aligner [17, 18]. We chose to use the STAR aligner due to its speed and ability to handle spliced reads, which occur in some viruses. Reads that did not align to the human genome were then mapped to a database of modified viral genomes (Fig. 1a).

Virus detection can be subject to poor specificity caused by areas of low complexity and sequence similarity to human sequences that are found in some viral genomes. To ameliorate this, the target viral genomes database was optimized to increase specificity by masking the viral genomes for (a) areas of human homology and (b) areas of low complexity (Fig. 1b). We used 93% nucleotide similarity across a sliding window of 75 nucleotides as evidence of homology. The masking step replaced nucleotides in these areas with Ns so that the aligner would not align any reads to the masked areas. This step addresses the problem of low complexity reads, which are abundant in RNA-seq data and can lead to false positive virus calls [6] (Fig. 1c). By performing in silico simulations of human and low complexity reads, we confirmed that masking the viral genome reduced the false positive rate from a median of 163/10^6^ for low complexity reads and 4.5/10^6^ for human simulated reads to a total of 2/10^8^ mapped reads for low complexity reads and 0/10^8^ human simulated reads.

We validated the performance of VirDetect using in silico simulations (see methods) of randomly drawn paired-end 50-mers from all virus genomes in our database that incorporated up to 10 base changes in the first read in the pair. For ≤3 mutations, the median sensitivity was 99.6% (Fig. 1d). For > 3 mutations, the sensitivity decreased linearly (Spearman’s rank correlation coefficient = − 0.96), down to a median of 23% for 10 random substitutions per 50 mer. The positive predictive value was 97% across all mutation levels (Fig. 1e), meaning that even when mutation burden was high, the specificity (virus reads mapping to the correct genome) remained high.

### Contamination in TCGA data as ascertained by VirDetect

**Fig. 2:**
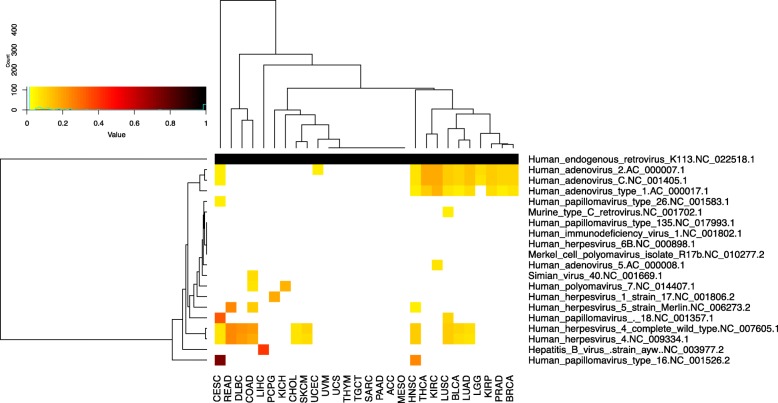
Prevalence of viral expression in TCGA. Viruses with > 5% prevalence in any TCGA cohort sequenced at UNC are shown on the y-axis and cancer types are on the x-axis. The color of each cell represents the proportion of samples with expression (> 2 reads) of each virus, if the prevalence is > 5%. Human endogenous retrovirus K113 was displayed as a positive control, since all samples have should express it

We assessed the extent of possible viral contamination by analyzing virally-derived reads in those TCGA samples that were sequenced at the University of North Carolina at Chapel Hill (all cancer types except glioblastoma, esophageal, gastric, acute myeloid leukemia and ovarian cancer, *n* = 9143, Additional file 1: Table S1, Fig. 2). As expected, hepatitis B virus (HBV) was prevalent (*n* = 152/368, 41%) in liver cancer. Our data were 83% concordant (true positive calls) to TCGA Research Network [19], which used consensus calls of different virus detection software and clinical data to identify HBV positive samples [13, 19, 20]. We did not find any hepatitis C virus sequences since TCGA RNA-seq used polyA selection and hepatitis C is not poly-adenylated [21]. HPV16 was prevalent in head and neck squamous cell carcinoma (HNSC) (> 0 reads, *n* = 125/495 (25%); > 1000 reads, *n* = 53/496 (10%)). Using > 0 reads, the concordance was 81% compared to TCGA Research Network [22], which used p16 immunostaining and in situ hybridization. Using the threshold of 1000 reads, as used by TCGA Research Network, HPV16 calls were completely concordant. HPV16 in cervical carcinoma (CESC) was present in 54% of samples (*n* = 163/301) and HPV18 was present in 15% (*n* = 44/301) of samples with > 1000 counts and was 99 and 96% concordant, respectively with TCGA Research Network’s HPV calls, which were RNA-seq based [23]. Thus, VirDetect detected the expected viruses in the appropriate tumor types.

**Fig. 3:**
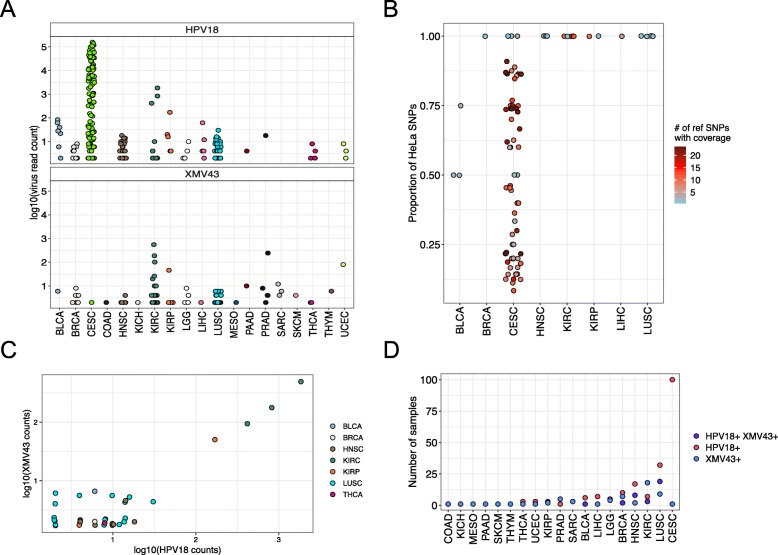
Expression of HPV18 and XMV43 in TCGA samples **a** Log10(virus read count) of HPV18 (top panel) and XMV43 (bottom panel) for all cancer types in TCGA that have expression of either virus. **b** Proportion of HeLa specific SNPs (defined in Cantalupo et al. [11]) that have a HeLa specific allele. The color of the dot represents the number of reference SNPs that had coverage across the HeLa allele. **c** Scatter plot of log10(HPV18 counts) on x-axis and log10(XMV43 counts) on y-axis for non-cervical cancer samples. The points are slightly jittered for due to overlapping points. **d** Number of samples that contain HPV18 (pink), XMV43 reads (blue), or both (purple) for cancer types with expression in either virus

Unlike the above noted viruses that we expected to observe in TCGA tissue, VirDetect also detected the presence of HPV18 in non-cervical cancer tumors, which is unlikely to be present. HPV18 sequences were found in 233 samples, 131 of which were non-cervical cancer samples. The median read count for HPV18 in non-cervical cancer samples was 4 with a maximum read count of 1836 (clear cell renal cell carcinoma (KIRC), sample: TCGA-CJ-5681). The mean read count for CESC samples was 14,298 reads, with a maximum read count of 156,772. HPV18 was also present in 9% of lung squamous cell carcinoma samples (LUSC, Figs. 2 and 3a) with a median read count of 4 and a maximum read count of 16. These order of magnitude differences suggested either an entirely different pathophysiology or contamination.

Cantalupo et al. found HPV18 in non-cervical samples to be derived from the HeLa cell line [11]. This finding was based on unique single nucleotide polymorphisms (SNPs) that were present in the genome of HPV18 in HeLa cells. Using the described 23 HeLa-specific SNPs, we found that except for CESC and three bladder cancer samples (described in the pathology reports as “invasion into the cervix”, possibly cervical cancer), all *n* = 17 non-cervical cancer samples that had coverage > 0 of these SNPs matched HeLa HPV18 strain completely (Fig. 3b), confirming what Cantalupo et al. previously found. This strengthens the hypothesis that the non-cervical HPV18 that was detected in TCGA samples was likely due to contaminating HeLa cells.

Ninety-six samples in TCGA had mRNA reads that aligned to an XMRV, specifically XMV43 (NC 001702.1, Murine type C), which was likely not present in any human tumor tissue, but resulted from demonstrated contamination in cell culture from an external source [5]. Notably, XMV43 had a median read count of 2, with a maximum read count of 554 in the same KIRC sample with the highest (non-cervical) expression of HPV18 (TCGA-CJ-5681, Fig. 3c). XMV43 was also present in 5% of LUSC samples and 3.5% of LUSC samples contained both XMV43 and HPV18 (Fig. 3d). The co-occurrence of these two unexpected viruses in the same sample suggested a common origin.

If HPV18 and XMV43 were introduced into the TCGA dataset as a result of contamination by a common event, e.g. at the same time, one would expect them to be present in the same samples and have correlated expression. For the samples with both XMV43 and HPV18, the expression was correlated (Spearman’s rank correlation coefficient = 0.44, *p* = 0.006, Fig. 3c). We then tested if HPV18 and XMV43 reads were present in the same samples more than expected by chance and found that they significantly co-occurred in breast cancer, HNSC, KIRC, renal papillary cell, and LUSC (both viruses were expressed in > 1 sample, FDR adjusted *p*-values, Fisher’s exact test, respectively: 0.03, 4.3 × 10^− 9^, 0.03, 0.01, 1.4 × 10^− 13^, Fig. 3d). Together, this indicates that the likely contaminant contained RNA from both viruses.

Among human cancers, second to CESC, HNSC is consistently associated with high risk human papillomaviruses; although, HNSC is very rarely associated with type HPV18 [24]. HPV18 and XMV43 reads did not significantly co-occur in CESC, even though CESC had the highest HPV18 positivity of all samples in the TCGA. The co-occurrence of HPV18 and XMV43 in HNSC, but not in CESC is consistent with the hypothesis that HPV18 and XMV43 were introduced into the sequencing pipeline together rather than originated from co-infected naturally occurring cancers.

### Investigations into the origin of the contamination

**Fig. 4:**
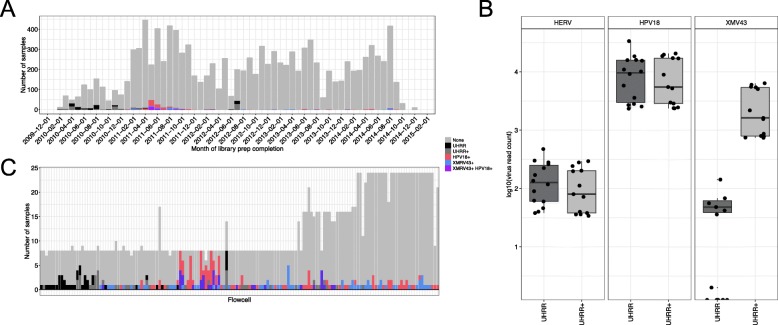
Viral expression across time. **a** Timeline of TCGA and UHRR library preparation. Stacked bar graph, with each bar representing one month. **b** Log 10 read counts of human endogenous retrovirus K113 (HERV, positive control), HPV18, or XMV43 in UHHR or UHHR+ samples. **c** Stacked bar graph showing the number of either UHRR or TCGA sample in each flowcell. The x-axis is organized by chronologically (oldest sample on left). Only flowcells that contained HPV18 (excluding CESC), XMV43, or a UHRR(+) sample were included. **a**&**c** The colors represent TCGA samples with no evidence of either HPV18, XMV43, or CESC samples (gray), samples with HPV18 reads (pink, excluding CESC samples), XMV43 reads (blue), non-cervical samples that contain both HPV18 and XMV43 (purple), or was a UHRR (black), or UHRR+ (dark gray) sample

To identify the root cause of contamination, each positive sample was investigated with respect to a shared event. The Stratagene Universal Human Reference RNA (UHRR, proprietary mixture of several cell lines) was sequenced in the same sequencing facility and contemporaneously with most of the TCGA samples to monitor the library preparation and sequencing procedures (Fig. 4a) [25]. Additionally, the lab stocks of two breast cancer cell lines, MCF-7 and ME16C, were added to the UHRR sequencing control sample to ensure that breast cancer gene expression was included in the human reference (will be referred to as UHRR+). Both UHRR and UHRR+ contained high levels of HPV18 transcripts, indicating that HeLa was likely included as one of the UHRR cell lines (Fig. 4b). By contrast, only the UHRR+ samples contained high levels of XMV43 transcripts. This suggests that one of the two additional cell lines was responsible for the presence of XMV43.

Most of the UHRR+ samples were sequenced in 2010, when none of the TCGA samples contained XMV43 or HPV18 reads (Fig. 4a). The evidence of UHRR+ contamination (i.e. HPV18 and XMV43 together) peaked in the spring/summer of 2011. Some samples with evidence of contamination did not have their library prepared on the same day as other UHRR(+) samples, meaning the presence of these viral sequences was not necessarily due to cross-contamination or “sample jumping” (RNA “jumping” to another tube due to static conditions) during library preparation. Also, “sample bleeding” was not observed due to several flow cells with only a single XMV43/HPV18 positive sample and sequenced on a different flowcell than a common reference sample (Fig. 4c, bottom panel).

The low levels of RNA from HPV18 and XMV43 may have only been observed due to an increase in sequencing depth. The sequencing depth in 2010 was lower than in 2011 by an average 20 million reads. The increase in sequencing depth corresponded to a change from the Illumina GAII to the Illumina HiSeq sequencer at the facility. The samples that contained a contaminant had a significantly higher number of reads than samples without a contaminant (*P* < 1 × 10^− 16^, Mann-Whitney *U-*test).

**Fig. 5:**
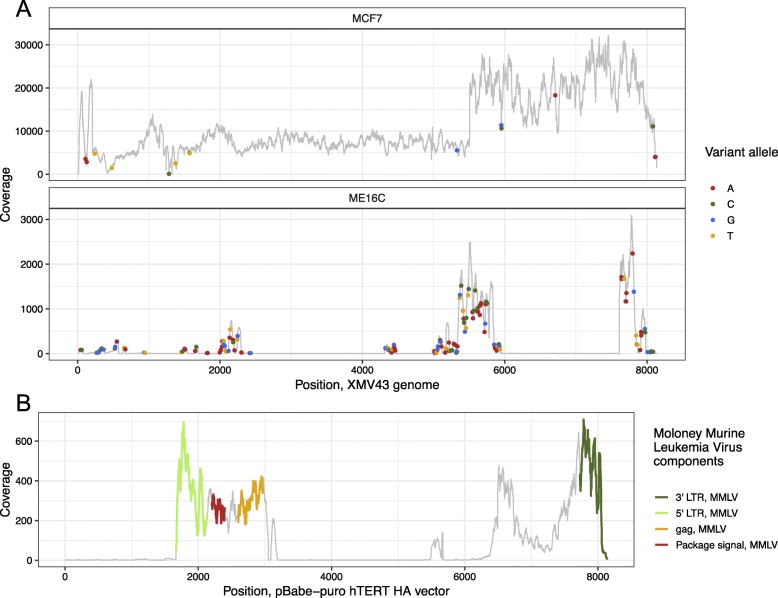
**a**&**b** Coverage plots. Position on the x-axis and coverage on the y-axis. **a** Alignments of laboratory stock of MCF-7 (top panel) and ME16C RNA-seq (bottom panel) aligned to the XMV43 genome. Dot represents a position with coverage > 10 and a variant allele with frequency > 0.9. The color of the dot represents the variant allele. **b** Alignments of ME16C RNA-seq to the pBabe-puro hTERT-HA vector sequence (addgene Plasmid #: 1772). The color on the sequence represents the different vector components that were derived from the Moloney Murine Leukemia Virus

Both of the lab stocks of MCF-7 and ME16C had pre-existing RNA-seq data (prepared on January of 2013). We detected the presence of XMV43-like sequences in both of these cell lines (Fig. 5a). MCF-7 had a higher abundance (1.8 × 10^6^ raw counts, 1% of total reads) compared to ME16C (1746 raw counts, 0.001% of total reads). The lab stock of MCF-7 had 21 nucleotides (nts, XMV43’s genome size is 8135 nts) compared to the reference XMV43 with an alternative allele frequency > 0.9 and ME16C had 160 nts with alternative allele frequencies > 0.9 with coverage >10X. Also, MCF-7 had >10X coverage across the entire genome in these samples, while ME16C had >10X coverage across just 40% of the XMV43 genome. The higher expression, higher sequence identity, and complete genome coverage of XMV43 in MCF-7 indicates that this cell line likely contributed to the XMV43 found in TCGA RNA-seq. To determine if the original MCF-7 cell line contained XMV43 or only this lab stock, we assessed publicly available RNA-seq of MCF-7, from Marcotte et al. (GSE73526) [26] and Qu et al. (GSE78512) [27]. The RNA-seq from both of these MCF-7 data sets contained no XMV43 reads. This suggests that XMV43 was only present in the laboratory stock of MCF-7 and not in the original cell line stock.

The incomplete alignments of ME16C sequences to XMV43-like were likely due to the presence of the pBabe-puro hTERT vector, which was used to transduce this cell line [28]. This vector contains mouse murine leukemia virus (MMLV) LTRs, packaging signal, and gag sequences, which contain low complexity regions of no significant sequence similarity to the human genome and thus were not masked by VirDetect. To differentiate virus-derived transcripts from viral-vector-derived transcripts, we added individual vector sequences to the VirDetect database. Assessing each component of the vector individually, as opposed to using UniVec [14], that contains the entire vector sequence, allowed for clearer resolution of what was transcribed. Many vectors in UniVec contain viral sequences (such as the human immunodeficiency virus and cytomegalovirus promoter/enhancer regions) and would increase false negative calls if all of UniVec was used as a filter. ME16C showed transcripts covering the puromycin resistance gene as well as the canonical SV40 promoter [29], which are both present in the pBABE-puro hTERT vector (Fig. 5b). The perfect alignments of MMLV elements to the vector and poor alignments to the XMV43 reference strongly suggests that XMV43 was detected in ME16C RNA-seq because of the vector used to transform the cell line. Together, this study elucidated a multistep contamination process. First, MCF-7 was infected with XMV43, which is known to infect human cells. Next, RNA from MCF-7 was added to the UHRR along with RNA from ME16C. This pool of RNA was sequenced alongside TCGA samples and became a low-level contaminant of the TCGA samples, although the specific event of how this contaminant was introduced remains unknown.

### Rabies virus expression, an additional signal of possible contamination

**Fig. 6:**
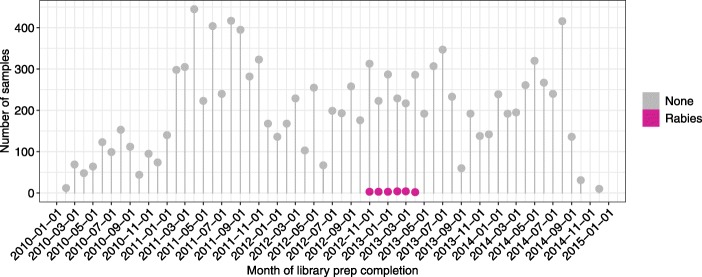
Timeline of TCGA library preparation showing the number of samples with rabies virus expression. The x-axis is the date of library preparation by every month, labeled every other month, and the y-axis shows the number of TCGA samples either with the expression of rabies virus or without for each month

We observed an additional virus signal in the RNA-seq that was likely due to laboratory contamination, however we were unable to determine the exact origin. We observed rabies virus expression with a read count of 2 in 19 samples from 10 different tumor types. These reads had high confidence alignments to rabies virus using BLAST as an independent verification step. Each of the samples had their libraries prepared from November of 2012 to April of 2013 (Fig. 6). Even though the virus was present at extremely low counts, the occurrence in adjacent time points suggests contamination.

## Discussion

Contamination in molecular biology has been a long and pervasive problem. RNA-sequencing is so sensitive that it can detect extremely low levels of contamination. Even with its ubiquity, contamination is a hazard to science, with the possibility of false positive claims and associations. We developed and validated a new virus discovery algorithm and database that allowed for high confidence in the virus calls. VirDetect can detect viruses with extremely high specificity because of the masked viral genomes.

From RNA-seq of tumor samples, differentiating natural virus infection from contamination is not always obvious and correlation with the presence of viral sequences alone is not evidence for causality. Some studies have used the criteria that a virus must have a certain expression threshold (mRNA levels) for the tumor to be virus-associated and that strength of association is therefore correlated with the strength of viral gene expression. This reasoning is sufficient for viruses and cancer types, where viruses are expected to be present in every single tumor cell, such as Epstein-Barr Virus (EBV) in EBV-associated gastric cancer or lymphoma [30]. It defines a conservative “gold standard”, but may miss situations, where the virus is present in only a fraction of the tumor cells or present in infiltrating, none tumor cells. These situations may never rise to the degree of establishing the virus in question as an etiological agent, but may nevertheless have utility in clinical decision making, e.g. in tumor classification or treatment selection. One such example is the established association of hepatitis B virus and liver cancer [16]. Another example is work by us and others of EBV transcripts in multiple cancers in the TCGA [31, 32]. These were well below the levels seen in clinically confirmed cases of gastric cancer and lymphoma and likely due to infiltrating lymphocytes, as we identified strong associations with B-cell abundance and altered B-cell receptor diversity.

RNA-seq contamination may arise from a PCR product, “sample jumping” (from tube to tube during laboratory handling of samples), “sample cross-talk” (read mis-assignment during pooling) [33, 34], or other possible technical phenomena that causes RNA or a read from one sample to be present in another. Being involved in producing most of TCGA’s RNA-sequencing, allowed us access to the laboratory metadata and enabled us to perform a forensic bioinformatics analysis. We confirmed the presence of HPV18 in non-cervical TCGA RNA-seq data and matched the SNPs to the specific HPV18 strain present in the HeLa cell line [11]. XMRV was found in the same samples as HPV18 more than expected by chance alone, indicating that the co-occurrence of both was likely due to the same exogenous contaminant.

In addition to these XMRV and Hela specific HPV contaminants, we also detected a small possible rabies virus contamination, albeit with very low read counts (2–19 total reads/contaminated sample). This strengthens the argument for the need for rigor and reproducibility in research, and to assist with this we provide VirDetect, as a robust tool for objective and accurate virus discovery and quantitation.

## Conclusions

Using RNA-seq and the laboratory metadata from TCGA, we were able to reconstruct the steps that lead to contamination. First the MCF-7 cell line was infected with an XMRV during local expansion, specifically XMV43. RNA from this cell line was then added to a pool of cell-line derived RNA (UHRR) that already contained HeLa. This pool of cell lines was sequenced contemporaneously and repeatedly with TCGA RNA-seq and during processing, a fraction of the TCGA sample RNAs were contaminated with the RNA from the standard pool of cell lines (UHRR+).

## Methods

### Virus detection, VirDetect

The VirDetect (https://github.com/dmarron/virdetect) database comprised of 1893 manually-curated vertebrate virus reference genomes from GenBank, downloaded on December 16, 2015. RNA-seq reads were aligned to hg38 (without chrEBV, which is an Epstein Barr Virus genome. Removed to enable detection of Epstein Barr Virus) using STAR v2.4.2a (1080 multi-maps, 10 mismatches). Unmapped reads were aligned to a masked viral FASTA using STAR v2.4.2a (52 multi-maps, 4 mismatches). Vertebrate viral FASTA (1894 viruses) was downloaded from GenBank and masked for increased specificity. All viruses were masked except for the human endogenous retrovirus K113 (NC_022518), which we used as a positive control. Regions were masked in two ways. (1) Viral reads of length 75 were simulated from the entire viral FASTA and then mapped to hg38 using STAR v2.4.2a (1080 multi-maps, 5 mismatches). If the viral simulated reads mapped to the human genome, they were masked in the viral FASTA. (2) Areas of low complexity (occurs in some viral genomes, 9 or more repeating single nucleotides (nts), 7 or more repeating double nts, 4 or more repeating nt patterns of 3, 3 or more repeating nts patterns of 4, 2 or more repeating patterns of 5, 2 or more repeating nt patterns of 6) were masked. Viruses were then quantified using the resultant SAM file. Vector component sequences were manually curated using available sequences at Vector Builder (https://en.vectorbuilder.com), AddGene (https://www.addgene.org), and Algosome (http://www.algosome.com/resources/common-sequences.html).

### In silico simulations

Scripts can be found here: https://github.com/sararselitsky/RNA-contamination-scripts. Random virus simulation: to simulate viral reads, a random virus and a random location within the virus were chosen. Fifty nts after that location comprised the first read in the pair. Then after a space of 200 nts, then the next 50 nts were used for the second read in the pair. The second read was reverse transcribed. Next, 0–10 mutations were randomly chosen and added to the first read in the pair at a randomly selected location. For each number of mutations, there were 100 simulated samples, each containing 1000 simulated reads.

Human transcriptome simulation: Human reads were simulated by randomly choosing a transcript from an hg38 transcript file generated by RSEM. A random location within the transcript was chosen as the first location for the first paired-end read. Then after a space of 200 nts, the next 50 nts comprised the second read pair. The reverse complement was taken of the second read pair. 100 simulated samples with 1000,000 paired-end reads in each sample were made. Low complexity simulation: Low complexity reads were simulated by generating all combinations of patterns of 1 (all As, all Ts…), 2 (AT, GC, CT, …), and 3 (CAC,CAA,CCA,…). Low complexity reads from this pool were randomly chosen and a random number of mutations were added to the first read pair. The second read was a reverse transcribed version of the first read pair, but without the mutations. 100 simulated samples, each with 1000 reads were generated.

### Sequencing of the universal human RNA reference

The UHRR+ was generated by adding 0.3 μg mRNA from MCF7 and 0.3μg mRNA from ME16C2 per 100 μg Stratagene Universal Reference RNA (Cat#740000–41). This was added to increase coverage of genes expressed in estrogen receptor positive and estrogen receptor negative breast cancers. One μg of total RNA from either UHRR or UHRR+ was converted to cDNA libraries using the lllumina mRNA TruSeq kit (RS-122-2001 or RS-122-2002) following the manufacturer’s directions. Libraries were sequenced 48x7x48bp on the Illumina HiSeq 2000 as previously described [35]. FASTQ files were generated by CASAVA.

### Details about the Hela SNP analysis

Script can be found here: https://github.com/sararselitsky/RNA-contamination-scripts/blob/master/HPV18_from_HeLa.pl. To determine the proportion of HeLa specific HPV18 SNPs (Table 3 from Cantalupo et al. [11]) we calculated the alternative allele frequency from the selected SNPs. If the HeLa alternative allele proportion was > 0.5, then this was considered a “HeLa SNP”, otherwise a reference SNP. Since contamination mostly led to low levels of HPV18 reads in non-cervical cancer samples, we did not have a coverage or allele count threshold. We calculated how many of the HeLa specific SNPs had an alternative allele compared to the reference.

### Statistics

All plots, except Fig. 5, and statistical analyses were performed using R version 3.4.1. The packages used were *ggplot2, reshape2,* and *gplots.*

## Supplementary information

**Additional file 1.** Virus count table. The columns are in the following order: analysis ID, cancer type, and then raw virus counts for all viruses with counts >0 in at least one sample.

## Data Availability

Viral counts available as supplemental data. TCGA data available on dbGaP accession phs000178.

## Abbreviations

CESC: Cervical carcinoma
EBV: Epstein-Barr Virus
HBV: Hepatitis B virus
H-HPV18: HeLa-derived HPV18
HNSC: Head and neck squamous cell carcinoma
HPV18: Human papillomavirus strain 16
HPV18: Human papillomavirus strain 18
KIRC: clear cell renal cell carcinoma
LTR: Long terminal repeat
LUSC: lung squamous cell carcinoma samples
MMLV: mouse murine leukemia virus
TCGA: The Cancer Genome Atlas
UHRR: Universal Human Reference RNA
UHRR+: Universal Human Reference RNA with the addition of MCF-7 and ME16C RNA
XMRV: xenotropic murine leukemia virus-related
XMV43: xenotropic murine leukemia virus-related 43
